# Association of early blood-based biomarkers and six-month functional outcomes in conventional severity categories of traumatic brain injury: capturing the continuous spectrum of injury

**DOI:** 10.1016/j.ebiom.2024.105298

**Published:** 2024-08-26

**Authors:** Lindsay Wilson, Virginia F.J. Newcombe, Daniel P. Whitehouse, Stefania Mondello, Andrew I.R. Maas, David K. Menon, CENTER-TBI participants and investigators, Cecilia Ackerlund, Krisztina Amrein, Nada Andelic, Lasse Andreassen, Audny Anke, Anna Antoni, Gérard Audibert, Philippe Azouvi, Maria Luisa Azzolini, Ronald Bartels, Pál Barzó, Romuald Beauvais, Ronny Beer, Bo-Michael Bellander, Antonio Belli, Habib Benali, Maurizio Berardino, Luigi Beretta, Morten Blaabjerg, Peter Bragge, Alexandra Brazinova, Vibeke Brinck, Joanne Brooker, Camilla Brorsson, Andras Buki, Monika Bullinger, Manuel Cabeleira, Alessio Caccioppola, Emiliana Calappi, Maria Rosa Calvi, Peter Cameron, Guillermo Carbayo Lozano, Marco Carbonara, Ana M. Castaño-León, Simona Cavallo, Giorgio Chevallard, Arturo Chieregato, Giuseppe Citerio, Hans Clusmann, Mark Steven Coburn, Jonathan Coles, Jamie D. Cooper, Marta Correia, Amra Čović, Nicola Curry, Endre Czeiter, Marek Czosnyka, Claire Dahyot-Fizelier, Paul Dark, Helen Dawes, Véronique De Keyser, Vincent Degos, Francesco Della Corte, Hugo den Boogert, Bart Depreitere, Đula Đilvesi, Abhishek Dixit, Emma Donoghue, Jens Dreier, Guy-Loup Dulière, Ari Ercole, Patrick Esser, Erzsébet Ezer, Martin Fabricius, Valery L. Feigin, Kelly Foks, Shirin Frisvold, Alex Furmanov, Pablo Gagliardo, Damien Galanaud, Dashiell Gantner, Guoyi Gao, Pradeep George, Alexandre Ghuysen, Lelde Giga, Ben Glocker, Jagoš Golubović, Pedro A. Gomez, Johannes Gratz, Benjamin Gravesteijn, Francesca Grossi, Russell L. Gruen, Deepak Gupta, Juanita A. Haagsma, Iain Haitsma, Raimund Helbok, Eirik Helseth, Lindsay Horton, Jilske Huijben, Peter J. Hutchinson, Bram Jacobs, Stefan Jankowski, Mike Jarrett, Ji-yao Jiang, Faye Johnson, Kelly Jones, Mladen Karan, Angelos G. Kolias, Erwin Kompanje, Daniel Kondziella, Evgenios Kornaropoulos, Lars-Owe Koskinen, Noémi Kovács, Ana Kowark, Alfonso Lagares, Linda Lanyon, Steven Laureys, Fiona Lecky, Didier Ledoux, Rolf Lefering, Valerie Legrand, Aurelie Lejeune, Leon Levi, Roger Lightfoot, Hester Lingsma, Marc Maegele, Marek Majdan, Alex Manara, Geoffrey Manley, Hugues Maréchal, Costanza Martino, Julia Mattern, Catherine McMahon, Béla Melegh, Tomas Menovsky, Ana Mikolic, Benoit Misset, Visakh Muraleedharan, Lynnette Murray, Nandesh Nair, Ancuta Negru, David Nelson, Daan Nieboer, József Nyirádi, Matej Oresic, Fabrizio Ortolano, Olubukola Otesile, Aarno Palotie, Paul M. Parizel, Jean-François Payen, Natascha Perera, Vincent Perlbarg, Paolo Persona, Wilco Peul, Anna Piippo-Karjalainen, Matti Pirinen, Dana Pisica, Horia Ples, Suzanne Polinder, Inigo Pomposo, Jussi P. Posti, Louis Puybasset, Andreea Rădoi, Arminas Ragauskas, Rahul Raj, Malinka Rambadagalla, Isabel Retel Helmrich, Jonathan Rhodes, Sylvia Richardson, Sophie Richter, Samuli Ripatti, Saulius Rocka, Cecilie Roe, Olav Roise, Jonathan Rosand, Jeffrey Rosenfeld, Christina Rosenlund, Guy Rosenthal, Rolf Rossaint, Sandra Rossi, Daniel Rueckert, Martin Rusnák, Juan Sahuquillo, Oliver Sakowitz, Renan Sanchez-Porras, Janos Sandor, Nadine Schäfer, Silke Schmidt, Herbert Schoechl, Guus Schoonman, Rico Frederik Schou, Elisabeth Schwendenwein, Ranjit D. Singh, Charlie Sewalt, Toril Skandsen, Peter Smielewski, Abayomi Sorinola, Emmanuel Stamatakis, Simon Stanworth, Robert Stevens, William Stewart, Ewout W. Steyerberg, Nino Stocchetti, Nina Sundström, Riikka Takala, Viktória Tamás, Tomas Tamosuitis, Mark Steven Taylor, Braden Te Ao, Olli Tenovuo, Alice Theadom, Matt Thomas, Aurore Thibaut, Dick Tibboel, Marjolijn Timmers, Christos Tolias, Tony Trapani, Cristina Maria Tudora, Andreas Unterberg, Peter Vajkoczy, Egils Valeinis, Shirley Vallance, Zoltán Vámos, Mathieu van der Jagt, Joukje van der Naalt, Gregory Van der Steen, Jeroen T.J.M. van Dijck, Inge A. van Erp, Thomas A. van Essen, Wim Van Hecke, Caroline van Heugten, Dominique Van Praag, Ernest van Veen, Roel P.J. van Wijk, Thijs Vande Vyvere, Alessia Vargiolu, Emmanuel Vega, Kimberley Velt, Jan Verheyden, Paul M. Vespa, Anne Vik, Rimantas Vilcinis, Victor Volovici, Nicole von Steinbüchel, Daphne Voormolen, Peter Vulekovic, Kevin K.W. Wang, Eveline Wiegers, Guy Williams, Stefan Winzeck, Stefan Wolf, Zhihui Yang, Peter Ylén, Alexander Younsi, Frederick A. Zeiler, Veronika Zelinkova, Agate Ziverte, Tommaso Zoerle

**Affiliations:** a Division of Psychology, University of Stirling, Stirling, United Kingdom; b Division of Anaesthesia and PACE, Department of Medicine, University of Cambridge, Cambridge, United Kingdom; c Department of Biomedical and Dental Sciences and Morphofunctional Imaging, University of Messina, Messina, Italy; d Department of Neurosurgery, Antwerp University Hospital, Edegem, Belgium; e Department of Translational Neuroscience, Faculty of Medicine and Health Science, University of Antwerp, Antwerp, Belgium

**Keywords:** Traumatic brain injury, Blood biomarkers, GFAP, NFL, UCH-L1, Outcomes

## Abstract

**Background:**

Traumatic brain injury is conventionally categorised as mild, moderate, or severe on the Glasgow Coma Scale (GCS). Recently developed biomarkers can provide more objective and nuanced measures of the extent of brain injury.

**Methods:**

Exposure–response relationships were investigated in 2479 patients aged ≥16 enrolled in the Collaborative European NeuroTrauma Effectiveness Research in Traumatic Brain Injury (CENTER-TBI) prospective observational cohort study. Neurofilament protein-light (NFL), ubiquitin carboxy-terminal hydrolase L1 (UCH-L1), and glial fibrillary acidic protein (GFAP) were assayed from serum sampled in the first 24 h; concentrations were divided into quintiles within GCS severity groups. Relationships with the Glasgow Outcome Scale-Extended were examined using modified Poisson regression including age, sex, major extracranial injury, time to sample, and log biomarker concentration as covariates.

**Findings:**

Within severity groups there were associations between biomarkers and outcomes after adjustment for covariates: GCS 13–15 and negative CT imaging (relative risks [RRs] from 1.28 to 3.72), GCS 13–15 and positive CT (1.21–2.81), GCS 9–12 (1.16–2.02), GCS 3–8 (1.09–1.94). RRs were associated with clinically important differences in expectations of prognosis. In patients with GCS 3 (RRs 1.51–1.80) percentages of unfavourable outcome were 37–51% in the lowest quintiles of biomarker levels and reached 90–94% in the highest quintiles. Similarly, for GCS 15 (RRs 1.83–3.79), the percentages were 2–4% and 19–28% in the lowest and highest biomarker quintiles, respectively.

**Interpretation:**

Conventional TBI severity classification is inadequate and underestimates heterogeneity of brain injury and associated outcomes. The adoption of circulating biomarkers can add to clinical assessment of injury severity.

**Funding:**

10.13039/501100000780European Union 7th Framework program (EC grant 602150), 10.13039/501100007731Hannelore Kohl Stiftung, 10.13039/100018727One Mind, 10.13039/100009006Integra LifeSciences, Neuro-Trauma Sciences, 10.13039/501100000272NIHR Rosetrees Trust.

**Research in context**

**Evidence before this study**

Traumatic brain injury (TBI) has the highest incidence of common neurological disorders. Conventional classification of severity of TBI is based on the Glasgow Coma Scale (GCS), with over 90% of patients attending hospital graded as ‘mild’ injuries (GCS 13–15) while the remainder are ‘moderate’ GCS 9–12 or ‘severe’ (GCS 3–8). Despite the label, mild TBI can result in incomplete recovery on the Glasgow Outcome Scale Extended (GOSE); in some studies 50% or more of patients have GOSE <8 at six months. However, it is uncertain whether problems are due to brain injury or other factors.

Recently developed blood biomarkers are measures of the burden of injury that are more objective and nuanced than the GCS. Biomarkers can potentially be used to study exposure–response relationships with outcome on the GOSE. We searched the Pubmed database since inception to September 1st 2023 using the terms ((“traumatic brain injury” OR TBI OR “head injury”) AND (“Glasgow Outcome Scale” OR GOS OR GOSE)) AND (biomarkers). We identified 287 papers, of which 58 concerned common blood-based biomarkers. One paper described survival analyses across biomarker concentration quintiles, but a quantile-based approach has not been applied to exposure–response relationships between biomarkers and functional outcomes.

**Added value of this study**

We found dose–response relationships between biomarker concentrations and functional outcomes on the GOSE, and these were present in all GCS severity groups (GCS 13–15 with or without abnormality on CT, GCS 9–12, and GCS 3–8). There was a continuous spectrum of biomarker concentrations and impaired outcomes across GCS groups, and overlap at the borderline between them. Ceiling and floor effects, a prominent feature of the GCS total scores, are not observed on biomarker concentration distributions. Within the group with GCS = 3, biomarker concentrations allow separation between patients with 49–60% chance of a favourable outcome vs those with a 90% or more likelihood of an unfavourable outcome. The extent of brain damage defined by biomarker levels plays a role in outcome in patients with GCS 13–15, including not only those with positive CT findings, but also those with a negative CT. Increasing biomarker concentrations were associated with an increase in absolute proportion of incomplete recovery. Furthermore, unfavourable outcomes after GCS 13–15 were almost entirely confined to patients in higher biomarker quintiles.

**Implications of all the available evidence**

The conventional distinction between mild, moderate, and severe TBI is too coarse, and the use of the term ‘mild’ to describe the heterogenous group who have an initial GCS 13–15 should be discontinued. While the role of pathophysiology in chronic disability after TBI with a presenting GCS 3–12 is well-appreciated, the evidence that brain injury also plays an important role in disability after GCS 13–15 should be recognised in management. Decisions in the acute stage concerning patients with low initial GCS scores should be informed by the range of outcomes achievable, including the likelihood of favourable outcome in patients with relatively low biomarker concentrations. Making biomarker assays available in routine clinical practice is an important future step for the field.

## Introduction

Traumatic brain injury (TBI) is frequently classified into severity bands using the Glasgow Coma Scale (GCS) score,^1^ with a broad division between so-called mild injuries (GCS 13–15) and moderate/severe injuries (GCS 3–12). The common perception of the term ‘mild’ is that brain injury is insignificant, has few or no negative consequences, and there is typically complete recovery.^2^ Recent work has challenged the latter concept: in some studies over 50% of patients with GCS 13–15 report disability at six months post-injury (defined as a score on the Glasgow Outcome Scale Extended [GOSE] <8).^3^ However, it is unclear to what extent persisting disability is related to brain injury, or to other influences on outcomes such as social and psychological factors or pre-existing conditions.^4^^,^^5^

Diffuse injury is a hallmark of TBI, in the form of diffuse axonal injury and/or multifocal brain lesions, and is often detectable on acute imaging, including in patients with GCS 13–15.^6^ However, imaging changes in the brain have proven difficult to summarise in a practical severity scale. Recently, available fluid biomarkers can provide a sensitive and objective measure of the overall burden of brain injury.^7^ Biomarkers are not influenced by prehospital management with sedation and intubation, or factors such as intoxication that affect the validity of the GCS as an assessment of severity.^8^

Recent studies have focussed on the prognostic value of biomarkers^9^, ^10^, ^11^ and their use in identifying distinct pathophysiological trajectories in the acute stage.^12^ However, considered as measures of severity of brain injury, biomarkers can also be used to address etiological questions concerning outcomes. For example, a biological gradient between biomarker concentrations and later functional outcomes would provide evidence for a role of brain injury in disability. Based on biomarkers identified in earlier studies with the greatest incremental value in prognosis, we selected three candidate measures for extent of injury that are relatively unaffected by extracranial injuries: neurofilament protein-light (NFL), ubiquitin carboxy-terminal hydrolase L1 (UCH-L1), and glial fibrillary acidic protein (GFAP). NFL is a marker of neuroaxonal injury related to long-term outcome.^13^ UCH-L1 is a protein expressed in neurons, and was identified in a previous study as the biomarker with the greatest incremental value in predicting the GOSE.^9^ GFAP is an astroglial marker that is strongly associated with early CT abnormality irrespective of type of lesion, and is also a predictor of outcome.^13^, ^14^, ^15^, ^16^

We employed data from the large-scale CENTER-TBI study to visualise exposure–response relationships using a quantile-based approach. We studied conventionally defined GCS severity groups: patients with GCS 13–15 divided into those with and without abnormalities on early CT, GCS 9–12, and GCS 3–8. The cohort has a relatively high proportion of patients with so-called complicated mild TBI, making it feasible to study these patients as a separate group.^6^ Contrary to the view that severity categories are distinct, we hypothesised that there would be heterogeneity within groups and continuity and overlap of biomarker values and outcomes between groups. The first objective was therefore to examine biomarker–outcome relationships within each of the four groups. Furthermore, we were interested in two specific issues: firstly the extent to which outcomes in patients with GCS 15 are associated with evidence of brain injury, and secondly whether biomarkers could reveal differences in outcome expectations among patients with an initial GCS of 3. The second objective was thus to examine relationships at each end of the GCS.

## Methods

### Participants

CENTER-TBI is a prospective observational cohort study that enrolled 4509 participants between 19th December 2014 and 17th December 2017. The rationale for the CENTER-TBI study and the sample size is provided in the project protocol.^17^ Inclusion criteria were: presentation within 24 h of TBI, referral for CT, and availability of informed consent for the study. Patients were excluded if they had a severe pre-injury neurological condition that would make outcome assessment problematic. Patients were recruited in three care pathways: evaluated in the emergency room and discharged (ER), admitted to hospital, and admitted to intensive care (ICU). The analysis included patients aged 16 or over who had biomarkers sampled in the first 24 h after injury and had a 6-month GOSE.

### Ethics

Ethical approval was obtained for each site in keeping with national and local regulations. The list of all sites, ethical committees granting approval and approval numbers can be found on the CENTER-TBI website (https://www.center-tbi.eu/project/ethical-approval). Written consent for participation was obtained at the earliest opportunity, but patients could be enrolled initially with oral consent.

### Clinical and demographic information

Information recorded by investigators at enrolment included demographic details, including the sex of the study participants, cause of injury, and pre-existing systemic disease on the American Society of Anaesthesiologists’ (ASA) classification of Physical Health.^18^ Conventional measures of severity of injury included early CT imaging,^19^ the Abbreviated Injury Scale (AIS) and Injury Severity Score (ISS)^20^ and GCS at baseline.^1^^,^^21^ Major extracranial injury (MEI) was defined as any non-head and neck AIS ≥3 (serious injury). Marshall CT classification categories are: (I) no visible intracranial pathology on CT, (II) cisterns present with any midline shift (MLS) <5 mm (III) cisterns compressed or absent with MLS <5 mm, (IV) MLS >5 mm, no mass lesion >25 cc, (V–VI) Mass lesion >25 cc.

### Global outcome

GOSE: The GOSE is an eight-category assessment of functional outcome ranging from death to complete recovery. The GOSE was assessed by interview^22^ or questionnaire,^23^ administered to a patient or carer, and then scored centrally. Since the vegetative state is not assessed separately by the questionnaire, this category was included with lower severe disability in the score. The GOSE rating emphasises change in function from pre-injury to post-injury and encompassed disability arising from all aspects of the injury, including extracranial injury. The GOSE largely captures the impairments detected by other outcome instruments^4^ and provides a rational basis for selection of additional outcome instruments in individual patients.^24^ For analysis, the GOSE was dichotomised as GOSE <8 (GOSE 2–7 vs 8), GOSE ≤6 (GOSE 2–6 vs 7–8), and GOSE ≤4 (GOSE 2–4 vs 5–8). Use of dichotomised outcomes is common in clinical trials in TBI and has been employed previously in studies of biomarkers.^9^^,^^10^ A six-month timepoint was chosen because it is a conventional timing for TBI outcomes, and it was the last timepoint at which all participants were scheduled for follow-up. When the follow-up was outside the protocol time window for six-month assessment, and assessments were available at other time points up to 18 months post-injury, missing GOSE values were imputed at 180 days using a multi-state model.^25^ For the cohort analysed a GOSE was available at 6 months for 2110/2479 (85%), and 369 (15%) were imputed from other timepoints.

### Biomarkers

Biomarker concentrations were measured from serum. The method of collection of samples and analysis of biomarker concentrations in CENTER-TBI has been described in detail elsewhere.^14^ Briefly, blood samples were collected using gel-separator tubes. After 45 ± 15 min of coagulation at room temperature, blood was centrifuged and aliquoted (8 × 0.5 mL). The samples were frozen at −80 °C and stored temporarily locally on site. Batches of samples were shipped on dry ice to the central CENTER-TBI biobank (Pecs, Hungary) and stored pending biochemical analysis. GFAP, NF-L and UCH-L1 were analysed using the Single Molecule Arrays (SiMoA) based Human Neurology 4-Plex B assay (N4PB) run on the SR-X benchtop assay platform (Quanterix Corp, Lexington, MA). The current study used a sample time window of 24 h.^15^ When more than one concentration was recorded then the highest value was used. NFL had an assay range of 0.0971–2000 pg per millilitre (pg/mL), UCH-L1 had a range of 1.34–40,000 pg/mL, and GFAP a range of 1.32–40,000 pg/mL. The lower limit of detection (LLOD) in pg/mL was 0.0971 for NFL, 1.34 for UCH-L1, and 1.32 for GFAP. Measured concentrations above and below the range were set to the maximum and minimum assay values, respectively. There were 6 values of NFL and 5 values of GFAP above the range, while there were 11 values of UCH-L1 that were below the LLOD, and none of these were zero. A log 10 transformation was applied to biomarker concentrations for analysis. In repeat analyses of a subsample of assays the coefficient of variation for the log-transformed biomarkers ranged from 22 to 30%.^14^

### Statistics

Patients in four GCS severity groups were compared on demographic and clinical characteristics using chi-square tests for categorical variables, ANOVA for age, and the Kruskal–Wallis test for biomarkers. Relationships between GCS and biomarkers described previously^14^^,^^26^ are updated here for context. Distributions of log biomarker concentrations were plotted against GCS total scores as violin plots. Density plots were created for GCS Total scores and for biomarker concentrations in different GCS groups. Bivariate relationships among biomarkers and other variables were evaluated using Spearman correlations.

The objectives of the study were addressed by graphing biomarker–outcome relationships in each of the severity groups and by formally assessing associations using regression analysis.

Within each GCS severity group biomarker concentrations were divided by quintiles to allow exposure–response relationships to be visualised. Percentages in each quintile with CT abnormalities and impairment on dichotomised GOSE scores were calculated with 95% confidence intervals (CIs).^27^

Relationships between biomarkers and outcomes were evaluated with modified Poisson regression.^28^^,^^29^ We adjusted for potential confounding variables in analysis including sex, age (in 10 year bands),^30^^,^^31^ extracranial injuries,^32^ and time from injury to sample (in three categories).^30^ Since we were interested in biomarkers as indicators of the burden of brain injury, we did not include variables on the same injury severity pathway, such as GCS score, CT findings, and pupillary reactivity.^33^ The adjusted contribution of log biomarkers to binary outcomes (as adjusted RRs) was used as an overall summary of relationships. The computation of RR is less susceptible to sparse data bias than the odds ratio, and is the preferred measure of association for etiological studies.^33^^,^^34^ Values over 1 indicate a positive relationship with percentage of impaired outcomes. We interpreted RRs if 1 was excluded from the 95% confidence interval, while recognising that uncertainty exists around estimates.^35^ We also examined the distribution of biomarker levels and their relationship to outcome at the floor and ceiling of the GCS (i.e. GCS 3 and 15, respectively).

Analyses were conducted using IBM SPSS Statistics 28 and R version 4.2.3. Data were collected on an electronic case report form (Quesgen Systems, USA), and downloaded from the International Neuroinformatics Facility (INCF) Neurobot platform (database version 3.0).

### Role of funders

Funding bodies played no role in study design, data collection, analysis and interpretation of data, writing the report, or the decision to submit for publication.

## Results

### Demographic and clinical characteristics

Of the 4509 patients enrolled in CENTER-TBI, 2479 were included in the analyses (mean age 50 years [SD 20.2], 1699 males [69%]); the selection process is shown in Supplementary Figure S1. There were 629 patients with GCS 3–8 (‘severe injuries’), 222 with GCS 9–12 (‘moderate injuries’) and 1628 with GCS 13–15 (‘mild injuries’). The GCS 13–15 group consisted of 868 patients with a negative initial CT and 760 patients with a positive CT. The majority of participants had a baseline GCS score that was either at the top or bottom of the range: 1243 (50%) had a GCS of 15 and 305 (12%) a GCS of 3 (Supplementary Figure S2). The characteristics of patients in the four severity groups are given in Table 1 together with univariate comparisons. The severity groups differed on demographic variables, including age, sex, level of education, employment and marital status. As expected, there were systematic differences between groups on variables associated with injury severity: care pathway, cause of injury, CT abnormality, AIS scores and extracranial injuries.

**Table 1:** Characteristics of participants.

	Participants, No (%)			GCS 9–12 (N = 222)	GCS 3–8 (N = 629)	p
	All eligible patients (N = 2479)	GCS 13–15 and CT− (N = 868)	GCS 13–15 and CT+ (N = 760)			
**Age**						<0.0001
Mean (SD) years	50 (20.2)	48 (20.1)	54 (20.0)	52 (20.7)	47 (20.0)	
**Sex**						<0.0001
Female	780 (32)	315 (36)	240 (32)	70 (32)	155 (25)	
Male	1699 (69)	553 (64)	520 (68)	152 (69)	474 (75)	
**Highest level of education**						0.0007
Primary	350 (17)	120 (15)	129 (21)	31 (20)	70 (16)	
Secondary	702 (34)	259 (32)	198 (32)	57 (36)	188 (42)	
Training	391 (19)	159 (20)	119 (19)	31 (20)	82 (18)	
College	599 (29)	270 (33)	183 (29)	40 (25)	106 (24)	
Missing	437	197	131	63	183	
**Employment Status**						<0.0001
Working (full or part time)	1176 (52)	464 (56)	330 (47)	95 (47)	287 (56)	
Not working	188 (8)	72 (9)	57 (8)	14 (7)	45 (9)	
Retired	623 (28)	195 (23)	241 (34)	70 (35)	117 (23)	
Student/homemaker	263 (12)	102 (12)	74 (11)	24 (12)	63 (12)	
Missing	229	35	58	19	117	
**Marital status**						0.0031
Partnered	1231 (54)	419 (51)	405 (57)	118 (58)	289 (53)	
Previously partnered	345 (15)	131 (16)	121 (17)	27 (13)	66 (12)	
Single/other	719 (31)	278 (34)	187 (26)	60 (29)	194 (35)	
Missing		40	47	17	80	
**Care pathway**						<0.0001
Emergency room	528 (21)	455 (52)	69 (9)	2 (1)	2 (<1)	
Admitted to hospital	721 (29)	336 (39)	352 (46)	27 (12)	6 (1)	
Intensive care unit	1230 (50)	77 (9)	339 (45)	193 (87)	621 (99)	
**ASA Pre-injury physical health**^c^						0.011
Healthy patient	1358 (56)	501 (58)	395 (52)	104 (48)	358 (59)	
Mild systemic disease	813 (33)	271 (32)	275 (37)	79 (36)	188 (31)	
Severe systemic disease	265 (11)	89 (10)	84 (11)	34 (16)	58 (10)	
Missing		7	6	5	25	
**Cause of injury**						<0.0001
Road traffic accident	984 (41)	315 (37)	276 (37)	78 (36)	315 (52)	
Fall	1090 (45)	415 (48)	368 (50)	99 (46)	208 (34)	
Violence/assault	118 (5)	49 (6)	34 (5)	12 (6)	23 (4)	
Other	231 (10)	84 (10)	63 (9)	25 (12)	59 (10)	
Missing/unknown		5	19	8	24	
**CT imaging abnormality**						N/A
Absent	920 (39)	868 (100)		14 (8)	38 (7)	
Present	1446 (61)		760 (100)	168 (92)	518 (93)	
Missing/uninterpretable	113			40	73	
Marshall CT classification						N/A
I.	917 (39)	866 (100)		14 (8)	37 (7)	
II.	920 (39)		606 (80)	86 (47)	228 (41)	
III	88 (4)		16 (2)	11 (60)	61 (11)	
IV	18 (1)		3 (<1)	3 (2)	12 (2)	
V–VI	415 (18)		132 (17)	68 (37)	215 (39)	
Missing	121		3	40	76	
**Baseline GCS**						N/A
3–8	629 (25)				629 (100)	
9–12	222 (9)			222 (100)		
13	97 (4)	14 (2)	83 (11)			
14	288 (12)	109 (13)	179 (24)			
15	1243 (50)	745 (86)	498 (66)			
**Head & neck AIS**^a^						<0.0001
No injury/Minor injury	435 (18)	368 (42)	51 (7)	8 (4)	8 (1)	
Moderate injury	327 (13)	247 (29)	65 (9)	7 (3)	8 (1)	
Serious injury	616 (25)	221 (26)	358 (47)	18 (8)	19 (3)	
Severe injury	426 (17)	26 (3)	204 (27)	81 (37)	115 (18)	
Critical injury	675 (27)	6 (1)	82 (11)	108 (49)	479 (76)	
**Major extracranial injury**^b^						<0.0001
Absent	1590 (64)	680 (78)	534 (70)	140 (63)	236 (38)	
Present	889 (36)	188 (22)	226 (30)	82 (37)	393 (63)	
**GOSE at six months**						<0.0001
Dead	274 (11)	10 (1)	38 (5)	50 (23)	176 (28)	
2/3 Lower severe disability/vegetative state	241 (10)	23 (3)	49 (6)	27 (12)	142 (23)	
4 Upper severe disability	109 (4)	16 (2)	34 (5)	15 (7)	44 (7)	
5 Lower moderate disability	234 (9)	42 (5)	76 (10)	32 (14)	84 (13)	
6 Upper moderate disability	250 (10)	59 (7)	97 (13)	22 (10)	72 (11)	
7 Lower good recovery	466 (19)	193 (22)	185 (24)	28 (13)	60 (10)	
8 Upper good recovery	905 (37)	525 (66)	281 (37)	48 (23)	51 (8)	
**Biomarkers**						
NFL median (IQR) pg/mL	20.8 (8.94–56.7)	8.78 (5.34–16.5)	21.6 (11.6–49.3)	48.2 (20.4–104)	63.5 (30.8–147)	<0.0001
UCH-L1 median (IQR) pg/mL	108 (39.5–354)	37.9 (18.9–73.0)	111 (51.6–274)	278 (129–624)	429 (203–903)	<0.0001
GFAP median (IQR) pg/mL	392 (65–1775)	43 (14–137)	505 (189–1480)	1600 (558–4450)	2230 (854–5210)	<0.0001
**Time to sample**						
NFL						<0.0001
0–8 h	769 (31)	410 (47)	183 (24)	46 (23)	130 (21)	
>8–16 h	704 (28)	213 (25)	213 (28)	65 (30)	213 (34)	
>16–24 h	1006 (41)	245 (28)	364 (48)	111 (47)	286 (46)	
UCH-L1						<0.0001
0–8 h	795 (32)	414 (48)	196 (26)	50 (23)	135 (22)	
>8–16 h	714 (29)	211 (24)	215 (28)	67 (30)	221 (35)	
>16–24 h	964 (39)	238 (28)	349 (46)	105 (47)	272 (43)	
Missing	6	5			1	
GFAP						<0.0001
0–8 h	774 (31)	410 (47)	187 (25)	47 (21)	130 (21)	
>8–16 h	708 (29)	213 (25)	214 (28)	64 (29)	217 (35)	
>16–24 h	997 (40)	245 (28)	359 (47)	111 (50)	282 (45)	

CT−, CT abnormality absent; CT+, CT abnormality present; NFL, neurofilament protein-light; neurofilament protein-light, UCH-L1, ubiquitin carboxy-terminal hydrolase L1; GFAP, glial fibrillary acidic protein; IQR, inter-quartile range.

N/A, Not applicable—the groups are different by design.

Statistical tests are for the difference between the four severity groups: chi-square tests for categorical variables, ANOVA for age, and the Kruskal–Wallis test for biomarkers.

a Head & neck AIS, maximum Abbreviated Injury Score for head, neck and cervical regions.

b Any non-head & neck AIS ≥3 (serious injury).

c ASA Pre-injury Physical Health, American Society of Anesthesiologists classification of physical health.

### Biomarker distributions and correlations

Median biomarker concentrations differed across groups, and increased as the severity of injury increased (Table 1 and Supplementary Figure S3). Concentration distributions for the GCS severity groups are shown in Supplementary Figure S4. Unlike the GCS distribution, there were no obvious ceiling or floor effects on biomarker concentrations in the total sample, or any of the GCS severity groups. Interval to sample was similar for the three biomarkers, and shortest for the group with GCS 13–15 and a negative CT (Table 1).

Biomarker concentrations were highly correlated with one another: GFAP and UCH-L1 r = 0.84 (0.82–0.87), NFL and UCH-L1 r = 0.76 (0.74–0.79), and NFL and GFAP r = 0.78 (0.75–0.80). Correlations with GCS for the three biomarkers were strong, albeit weaker than with one another: NFL (r = −0.55 [−0.59 to −0.52]), UCH-L1 (r = −0.60 [−0.64 to −0.57]), and GFAP (r = −0.60 [−0.64 to −0.57]). Age was moderately correlated with NFL concentration (r = 0.26 [CIs 0.22–0.30]), but was only very weakly associated with UCH-L1 (r = 0.05 [0.01–0.09]), GFAP (r = 0.05 [0.01–0.09]), and GCS (r = 0.07 [0.03–0.11]). The total extracranial ISS score correlated moderately with NFL (r = 0.24 [0.20–0.28]), UCH-L1 (r = 0.31 [0.27–0.34]), GFAP (r = 0.23 [0.19–0.27]), and also the GCS (r = −0.27 [−0.31 to −0.23]).

### Biomarkers and CT abnormalities

**Fig. 1:**
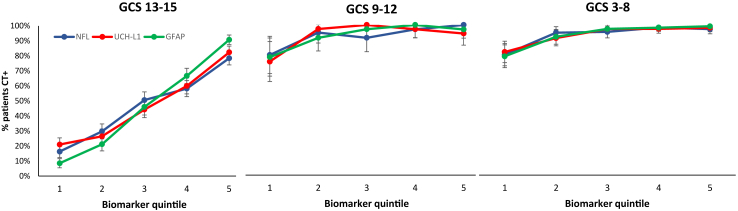
Percentages of patients with intracranial abnormality on early CT (CT+). Biomarker concentrations are divided into quintiles within each GCS severity group (GCS 13–15 N = 1628, GCS 9–12 N = 222, and GCS 3–8 N = 629). Error bars are 95% confidence intervals for percentages.

Percentages of patients with a CT abnormality in each biomarker quintile are shown in Fig. 1. Medians and ranges of concentrations for quintiles are given in Supplementary Table S1. As can be seen from Fig. 1, in the GCS 13–15 group the slopes for all three biomarkers showed almost linear relationships between concentrations and percent CT positive, confirming a close relationship between biomarkers and contemporaneous imaging. In the GCS 3–8 and 9–12 groups there is overlap with GCS 13–15 in the lowest quintiles. The trends for GCS 9–12 and 3–8 are similar to one another and are attenuated as they reach ceiling at upper concentrations of biomarkers.

### Biomarkers and outcome

**Fig. 2:**
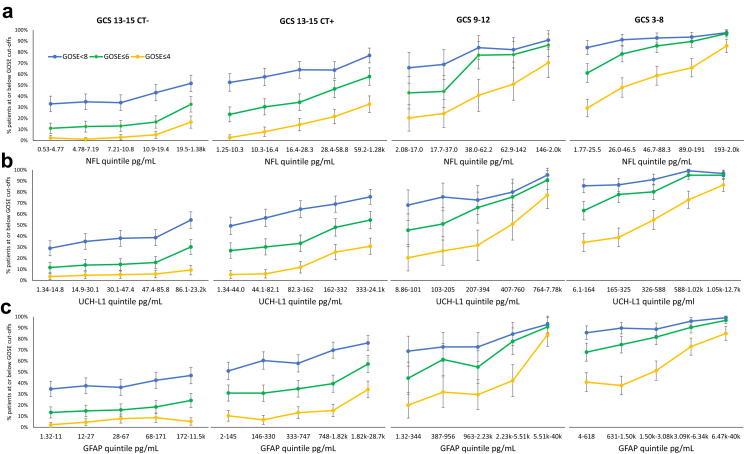
Biomarker exposure–response relationships for the 6-month GOSE. Biomarker concentrations are divided into quintiles within each GCS severity group (GCS 13–15 CT− N = 868, GCS 13–15 CT+ N = 760, GCS 9–12 N = 222 and GCS 3–8 N = 629). The figure shows the four severity groups in columns and the biomarkers in rows: a NFL, b UCH-L1 and c GFAP. The graphs show percentages of patients at or below three GOSE cut-offs (<8, ≤6, ≤4) in each quintile. Error bars are 95% confidence intervals for percentages.

Fig. 2 shows exposure–response relationships between biomarker concentrations and dichotomised six-month outcomes (source data in Supplementary Table S2). The overall trajectories of impaired outcomes for the biomarkers are similar, and appear to have lower asymptotes in the GCS 13–15 CT negative group and then extend across the range of concentrations to the highest quintile in GCS 3–8. There is overlap between each of the severity groups in percentages of impaired GOSE scores, particularly between the upper biomarker quintile of one group and the lowest biomarker quintile of the succeeding group (Fig. 2).

Levels of impairment in the lowest biomarker quintile varied across level of dichotomisation and were similar for the three biomarkers (Fig. 2, Supplementary Table S2). For GCS 13–15 with a negative CT, the first quintile percentage of GOSE <8 was 29–35% (50/172–62/179), GOSE ≤6 was 11–13% (19/172–24/179), and GOSE ≤4 was 2–3% (4/179–6/172) for the three biomarkers. In the highest quintile of the GCS 13–15 CT positive group, the percentage GOSE <8 was 76–77% (116/152–117/152), GOSE ≤6 was 55–58% (83/152–88/152), and GOSE ≤4 was 31–34% (47/152–52/152). Corresponding figures for the highest quintile in the GCS 3–8 group were 97–99% (121/125–125/126), 95–97% (119/125–122/126), and 85–86% (107/126–108/125), respectively. Dichotomisation at GOSE <8 and GOSE ≤6 appeared to be at or close to ceiling at higher biomarker levels in the GCS 3–8 group.

In the regression analyses the covariates age, sex, major extracranial injury and time to sample were associated with outcomes in individual analyses, with age having the most consistent relationship followed by major extracranial injury (Supplementary Tables S3–S5).

The adjusted RRs for associations between biomarkers and outcomes are given in Table 2 (unadjusted RRs are provided in Supplementary Table S6). RRs are expressed per log unit of biomarker concentration (for reference, log units for quintiles are provided in Supplementary Table S1). For patients with GCS 13–15 and a positive CT the adjusted relative risks (RRs) were greater than 1 for all GOSE cut-points and all biomarkers (RRs for GOSE <8 from 1.21 to 1.29; GOSE ≤6 from 1.36 to 1.72; and GOSE ≤4 from 2.14 to 2.81; all 95% CIs excluded the value 1), while in patients with a negative CT the RR was greater than 1 for NFL and UCH-L1 (GOSE <8 1.28 and 1.28; GOSE ≤6 1.97 and 1.71; and GOSE ≤4 3.72 and 1.84, respectively), but not for the association with GFAP. For patients with GCS 9–12 RRs were greater than 1 for UCH-L1 and GOSE <8, and for all biomarkers and GOSE ≤6 (RRs 1.28–1.39) and GOSE ≤4 (RRs 1.55–2.02). As can be seen in Fig. 2, the CIs for this group are broad, reflecting the relatively small number of cases who were GCS 9–12 in comparison to other severity groups. For patients with GCS 3–8 the adjusted RRs were again greater than 1 for all cut-points and all biomarkers (RRs for GOSE <8 from 1.09 to 1.12; GOSE ≤6 from 1.25 to 1.36; and GOSE ≤4 from 1.56 to 1.94). There were thus associations between biomarkers and outcomes within each severity group, a finding that extended to patients with GCS 13–15 and a negative CT.

**Table 2:** Adjusted relative risks (RRs), with 95% confidence intervals in brackets, for the association between log biomarker concentrations and outcomes within each GCS severity group (GCS 13–15 CT− N = 868, GCS 13–15 CT+ N = 760, GCS 9–12 N = 222 and GCS 3–8 N = 629).

	RR (95% CI)	p	RR (95% CI)	p
	GCS 13–15 CT−		GCS 13–15 CT+	
GOSE <8				
Log NFL	1.28 (1.05–1.58)	0.016	1.29 (1.15–1.44)	<0.0001
Log UCH-L1	1.28 (1.09–1.51)	0.0031	1.33 (1.21–1.47)	<0.0001
Log GFAP	1.00 (0.88–1.14)	0.99	1.21 (1.12–1.31)	<0.0001
GOSE ≤6				
Log NFL	1.97 (1.43–2.73)	<0.0001	1.72 (1.44–2.04)	<0.0001
Log UCH-L1	1.71 (1.29–2.26)	0.0002	1.64 (1.40–1.91)	<0.0001
Log GFAP	1.07 (0.85–1.35)	0.58	1.36 (1.19–1.56)	<0.0001
GOSE ≤4				
Log NFL	3.72 (2.47–5.60)	<0.0001	2.81 (2.14–3.68)	<0.0001
Log UCH-L1	1.84 (1.09–3.12)	0.023	2.92 (2.19–3.90)	<0.0001
Log GFAP	1.03 (0.69–1.54)	0.89	2.14 (1.69–2.70)	<0.0001
	GCS 9–12		GCS 3–8	
GOSE <8				
Log NFL	1.11 (0.97–1.26)	0.14	1.09 (1.04–1.14)	0.0003
Log UCH-L1	1.16 (1.04–1.30)	0.011	1.12 (1.06–1.19)	0.0001
Log GFAP	1.07 (0.98–1.17)	0.12	1.09 (1.04–1.13)	0.0001
GOSE ≤6				
Log NFL	1.39 (1.16–1.67)	0.0003	1.29 (1.20–1.39)	<0.0001
Log UCH-L1	1.49 (1.27–1.76)	<0.0001	1.36 (1.25–1.48)	<0.0001
Log GFAP	1.28 (1.11–1.48)	0.0006	1.25 (1.16–1.34)	<0.0001
GOSE ≤4				
Log NFL	1.78 (1.40–2.28)	<0.0001	1.68 (1.50–1.89)	<0.0001
Log UCH-L1	2.02 (1.58–2.58)	<0.0001	1.94 (1.68–2.24)	<0.0001
Log GFAP	1.55 (1.23–1.95)	0.0002	1.56 (1.38–1.77)	<0.0001

The RRs have been adjusted for age, sex, major extracranial injury, and time to sample.

As can be seen from Table 2, for each dichotomisation threshold, RRs were generally similar for all three biomarkers. An exception is GFAP which, after adjustment, showed little or no association with outcome in the group with GCS 13–15 and a negative CT. Within each severity group the size of the RRs generally increased as the level of dichotomisation changed from GOSE <8 to GOSE ≤4.

**Fig. 3:**
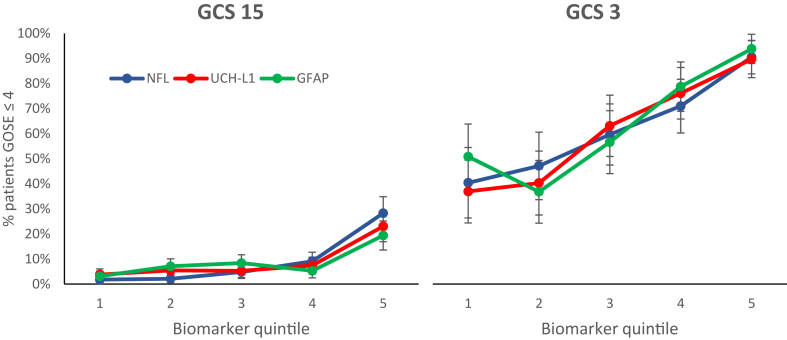
Biomarker exposure–response relationships for unfavourable outcome (GOSE ≤4) in patients with GCS 15 (N = 1243) and GCS 3 (N = 305). Quintiles for GCS 15 and GCS 3 are from the GCS 13–15 and GCS 3–12 groups, respectively. The graphs show percentages of patients at or below the GOSE cutoff in each quintile for NFL, UCH-L1 and GFAP. Error bars are 95% confidence intervals for percentages.

In each of the subgroups at floor and ceiling on the GCS (i.e. GCS 3 and 15, respectively), there was a spread of biomarker values, with overlap between the two subgroups for all three biomarkers (Supplementary Figure S3). In each category, the biomarkers showed dose response-relationships, with RRs for unfavourable outcome of 1.83–3.79 for GCS 15, and 1.51–1.80 for GCS 3 (Fig. 3, Supplementary Table S7). These RRs resulted in clinically significant expectations of prognosis (Supplementary Table S8). While the overall expectation of death or disability in the GCS 3 population was 65% (197/305), this dropped to 37–51% (17/46–29/57) in the lowest quintiles of biomarker levels, and reached 90–94% (70/78–61/65) in the highest quintiles. Differences were also noted for the GCS 15 cohort, with an overall expectation of unfavourable outcome of 8% (97/1243), but separating to 2–4% (5/277–11/287) and 19–28% (33/170–51/80) in the lowest and highest biomarker quintiles, respectively.

## Discussion

### Continuous vs categorical approaches to characterisation of TBI severity

The findings show that there is a continuous spectrum of injury severity extending from the lowest biomarker concentrations in GCS 13–15 to the highest in GCS 3–8. There are similar trends for all three biomarkers, thus providing converging evidence for the dose–response patterns observed. Furthermore, there is overlap between the GCS severity groups in biomarker concentrations, prevalence of CT abnormalities, and percentages of impaired outcomes, particularly between the upper biomarker quintile each group and the lowest biomarker quintile of the adjoining group. There is thus no clear boundary between GCS 13–15 and GCS 9–12 that defines a ‘mild’ injury. Furthermore, the GCS 9–12 group overlaps substantially with the GCS 3–8 group, suggesting that the distinction between moderate and severe injuries carries little meaning as far as outcomes are concerned.

While the findings argue in favour of a continuous view of severity, the division between GCS 13–15 and GCS 3–12 may be considered convenient in research studies and clinical practice. As apparent in Fig. 2, there is progression from lower levels of biomarkers in GCS 13–15, where GOSE <8 is a predominant impairment and GOSE ≤4 is rare, to the highest levels in GCS 3–12, where GOSE <8 is at ceiling and GOSE ≤4 is common. These overall differences in outcomes between GCS groups are well recognised in past work, and provide one rationale for separating groups in clinical trials or observational studies. The outcomes that are feasible and relevant to assess in patients with lower and higher degrees of disability are different.^24^ Arguing against broad distinctions is heterogeneity within each severity group. Regardless of whether the categories are convenient, the terminology is too crude to describe individual patients.

The presence of ceiling and floor effects on the GCS may have encouraged the view that TBI severity is bimodal. In contrast, biomarker concentration distributions appear unimodal, with no evidence of ceiling or floor effects. The use of the GCS as a measure of severity may contribute to undue optimism concerning recovery in patients with a high score and undue pessimism concerning prognosis with a low score.^36^ Low scores may reflect not just severity of the primary injury, but also alcohol or drug intoxication, post ictal unresponsiveness, or the effect of drugs used for emergency endotracheal intubation.^8^ These confounders of a very low GCS do not have the same prognostic import as an unconfounded low GCS, but may still affect decisions regarding advanced therapies such as decompressive craniectomy (in severe intracranial hypertension) or extracorporeal membrane oxygenation (in severe extracranial injury with refractory hypoxaemia), where elevated biomarkers may increase probability of prognostic defining lesions including the brain stem.^37^ While the overall expectation of death or disability in the GCS 3 cohort was ∼65%, biomarker measurements allowed separation of patients, with expectations of unfavourable outcomes in the lowest biomarker quintile dropping to 37–51%, and that in the highest quintile rising to 91–94%. This clear refinement of outcome expectations provided by blood biomarkers could inform discussions with families, provide better decision making regarding aggressive ICP-directed or systemic interventions, and temper decisions regarding early withdrawal of life sustaining therapies.

Past support for a broad distinction between GCS 13–15 and GCS 3–12 has come from work on prognostic models, and specifically the finding that predictors differ in the two severity groups.^38^ However, studies of prognosis have often focussed on GOSE <8 in patients with GCS 13–15 and on GOSE ≤4 in patients with GCS 3–12. Biomarker studies have consistently found stronger associations with GOSE ≤4 than GOS <8.^9^^,^^10^^,^^39^ In keeping with these reports, we found consistently larger RRs for GOSE ≤4 than GOSE <8 within the severity groups. An implication is that there are a wider range of influences on GOSE <8 and impairment is less predictable from biomarkers than for GOSE ≤4. Nevertheless, the continuous association of biomarker quintiles with outcome argues for effects of microstructural damage on outcome.

### The role of brain injury in outcome after GCS 13–15

The study provides evidence of a substantial burden of brain injury within the GCS 13–15 group. The relationship is clearest in the CT positive group, where percentages of impairment on the GOSE show a regular increase with increasing biomarker concentrations. In the top GCS 13–15 quintile, around three quarters of patients had GOSE <8, over half were GOSE ≤6, and 30% or more were GOSE ≤4. There would be value in using biomarkers to identify patients who would benefit from screening for impairment risk and initiation of early intervention.^40^

In the CT negative group there is also evidence of an association of outcome with biomarkers. While the relationship appears less systematic than for patients with positive CT, it is important because the association indicates that CT occult lesions play a role in outcome. A previous study examined the relation between outcome and NFL and GFAP in 55 patients with a negative CT, but did not find an association.^16^ However, our finding is concordant with work showing associations between MR imaging and outcome in patients with GCS 13–15 and a negative CT.^41^^,^^42^ While findings are present for NFL and UCH-L1, we did not find an association with GFAP, suggesting it may be less useful than the other biomarkers in this context. At the lower end of the biomarker range, variability in concentration measurement is an issue, and this will tend to attenuate relationships. A further limitation of the analyses is that the numbers of events may be low in some comparisons at the end of the range, and sparse data may contribute to the lack of observed associations.^43^

A feature of the CT negative group is the presence of lower limits or asymptotes for the prevalence of impairment on the GOSE in the lower concentration quintiles: GOSE <8 apparently has a lower limit at around 30% impaired, while for GOSE ≤6 it is approximately 15%, and for GOSE ≤4 it is around 3%. These observations are consistent with earlier reports of persisting limitations in patients with a negative CT in the TRACK-TBI study.^44^^,^^45^ Persisting disability may have origins in non-TBI factors including pre-existing disability, extracranial injuries, problems with mental health and emotional adjustment after trauma. The evidence from MR studies suggests that brain injury also plays a role.^41^^,^^42^ Furthermore, there are brain changes such as chronic inflammation^46^ that are not encompassed by the biomarkers included in the study. The presence of reported disability in this group cautions against applying the term ‘mild’ even at low levels of biomarkers.

Previous studies of the prognostic value of biomarkers have found that relatively little is added to prediction of incomplete recovery in patients with GCS 13–15.^9^^,^^10^ This is consistent with the curves shown in Fig. 2 for GOSE <8 in the two GCS 13–15 groups: these have relatively shallow slopes, origins above zero, and do not approach 100% impairment in this group. Biomarker concentrations indicate the probability of different levels of impairment, but do not predict individual outcomes with accuracy. Nonetheless, there are substantial changes in percentages of impaired outcomes across the biomarker range: increasing biomarker levels were associated with an increase in absolute prevalence of GOSE <8 by 24–26% in the GCS 13–15 CT positive group. Similar observations concerning the magnitude of change can be made for GOSE ≤6 and GOSE ≤4. The findings indicate that GOSE ≤4 at six months will almost always be associated with increased biomarker concentrations, since in the lowest biomarker quintile in the CT negative group the prevalence of an impaired outcome was only 1–3%. In the acute stage there would be uncertainty in predicting outcome, but at six months the early biomarker findings would afford high confidence in attributing brain injury a major role in disability. Judgements about likely causes of disability are central to the clinical management of patients in the later stages of recovery, and early biomarkers can play a crucial role in this context.

The extent of brain injury in individuals with GCS 13–15 is heterogeneous with respect to both biomarkers and CT abnormalities, implying that better characterisation of injury in individuals is needed. For patients the description ‘mild’ may lead to their problems being attributed to factors other than brain injury and difficulties in accessing appropriate care and resources.^47^ Biomarkers can make a contribution to identifying brain injury at the microstructural level. A key step will be implementation of uniform methods of collecting biomarkers, and consensus on their use to define severity of injury after adjusting for factors such as age. Furthermore, biomarkers need integrated with other clinical measures of injury severity, such as GCS and CT.^11^

### Strengths and limitations

The study has a number of strengths, including the large sample size, detailed characterisation of acute clinical characteristics, availability of recently developed blood biomarkers, and systematic follow-up for outcomes. Nonetheless there are limitations that should be borne in mind.

Caution needs to be exercised in relation to severity of injury and levels of impairment in the groups with GCS 13–15. Recruitment to the study was in specialist neuroscience centres in Europe and required that patients were triaged to CT. A relatively high proportion of patients with GCS 13–15 had a positive CT scan: around 50% compared to 5–10% typically reported.^48^^,^^49^ CENTER-TBI was an observational study conducted mainly in level 1 trauma centres that was designed to enrol patients in three care pathways (Table 1), and 79% of patients were admitted to hospital or intensive care. Thus the group studied here is enriched for so-called complicated mild TBI,^6^ and while this provides an opportunity for study, it means that care should be taken generalising findings to other settings. Similarly, missing data in CENTER-TBI, which is common in TBI research, potentially represents a limitation to generalisation.^24^^,^^50^

The current study was limited to functional recovery on the GOSE. The GOSE consists of broad categories of disability, and detailed information concerning symptoms and impairments is not recorded. The nature of the scale limits the kinds of analysis that can be applied to the data. Factors that affect development of disability, such as psychosocial influences, were not included in the study. A further element is information concerning localisation of injury in the brain. The combination of biomarkers and brain imaging represents an important avenue for future research,^11^ and is likely to prove critical to characterising injury in the individual. Investigation of biomarkers in relation to different dimensions of outcome, including patient-reported outcomes, is the subject of a separate CENTER-TBI study.

In line with previous work,^7^ the findings suggest that, with a few exceptions, associations with the three biomarkers are similar. We did not carry out a systematic comparison of the biomarkers, and it is likely that detailed differences will exist between them. For example, NFL seems to show the greatest quintile to impairment scaling in GCS 15. The biomarkers studied have different kinetics and the choice of time window may affect findings. In the current study only around 30% of samples were collected within 8 h. Furthermore, the coefficients of variation were relatively high. The biomarkers included are related to particular substrates, and will not reflect all pathophysiology present after TBI, such as, for example, neuroinflammatory processes.^46^ Finally, we chose potential confounding covariates based on current knowledge in the TBI biomarker field. Ideally confounders would be based on a causal model, such as a directed acyclic graph.^34^ Other alternatives such as the disjunctive cause criterion could be considered for selection of confounders.^51^ It is also possible that there are unmeasured confounding factors that influence relationships.

### Conclusions

Conventional TBI severity classification is inadequate for description of individual patients. Patients with GCS 13–15 have heterogeneous brain injuries, and as a group, show relationships between impaired outcomes and biomarker concentrations, with substantial long-term disability in patients with high levels of biomarkers. The use of the term ‘mild’ to describe the broad category of patients with GCS 13–15 should be discontinued. In patients with GCS 3, biomarker levels may allow more refined prognostication of outcome, inform family discussions, and rationalise management decisions. As recently developed biomarkers become standardised and clinically available, they promise to capture the continuous spectrum of injury severity after TBI and add to better characterisation of the injury.

## Contributors

LW, VFJN, DPW, SM, AIRM, DKM participated in the concept, design, analysis, writing, or revision of the manuscript. LW and DKM had the original idea for the study. LW and DPW analysed the data. LW, VFJN, DPW, SM, AIRM, DKM participated in the interpretation of results. LW and DKM prepared the draft manuscript and coordinated its finalisation. All authors read and approved the final version of the manuscript. LW and DPW verified the underlying data. All authors had full access to study data, and LW and DKM had final responsibility for the decision to publish. CENTER-TBI participants and investigators were responsible for the conduct of the project as a whole.

## Data sharing statement

Individual participant data are available conditional on approved study proposal, with no end date. Data to achieve the aims in the proposal will be available to researchers who describe a methodologically sound study that is approved by the CENTER-TBI management committee. Proposals can be submitted online at https://www.center-tbi.eu/data. A data access agreement is required, and all access must comply with regulatory restrictions imposed on the original study.

## Declaration of interests

Data were obtained in CENTER-TBI, a large collaborative project with the support of the European Union 7th Framework program (EC grant 602150). Additional funding was obtained from the Hannelore Kohl Stiftung (Germany), from OneMind (USA) and from Integra LifeSciences Corporation (USA), and NeuroTrauma Sciences (USA). Data for the CENTER-TBI study has been collected through the Quesgen e-CRF (Quesgen Systems Inc, USA), hosted on the INCF platform and extracted via the INCF Neurobot tool (INCF, Sweden). VFJN holds a NIHR Rosetrees Trust Advanced Fellowship, NIHR302544, which is funded in partnership by the NIHR and Rosetrees Trust. DKM reports grants, personal fees, and non-financial support from GlaxoSmithKline outside the submitted work; personal fees from Neurotrauma Sciences, Lantmannen AB, PressuraNeuro, CSL Behring, and Invex Ltd, outside of the submitted work. Dr Maas reports receiving personal fees from NeuroTrauma Sciences, and PressuraNeuro outside the submitted work. VFJN reports a grant with ROCHE Pharmaceuticals outside the submitted work, and personal fees from Integra outside the submitted work. Dr Wilson reports receiving personal fees from Novartis, Neurotrauma Sciences, and Mass General Brigham outside the submitted work. No other disclosures were reported.

## Appendix A Supplementary data

Nonauthor Collaborators

GOSE and biomarkers Suppl
