# Supplementary material for: Association of early blood-based biomarkers and six-month functional outcomes in conventional severity categories of traumatic brain injury: capturing the continuous spectrum of injury
Source: eBioMedicine. 2024 Aug 26;107:105298. doi: 10.1016/j.ebiom.2024.105298 (PMC11400615; doi:10.1016/j.ebiom.2024.105298)
Supplement: GOSE and biomarkers Suppl [file mmc2.docx]

**Supplementary material for Wilson et al “Association of early blood-based biomarkers and six-month functional outcomes in conventional severity categories of traumatic brain injury: capturing the continuous spectrum of injury”**

Supplementary Figure 1. CONSORT diagram for analysis sample

Supplementary Figure 2. Distributions of baseline GCS total scores

Supplementary Figure 3. Violin plots of log biomarker concentrations

Supplementary Figure 4. Distributions of concentrations of log NFL, log UCH-L1 and log GFAP

Supplementary Table 1. Number of samples (N), median, range (minimum and maximum value) and log 10 of the median for biomarker concentrations

Supplementary Table 2. Number of impaired scores on the dichotomised GOSE for each quintile band

Supplementary Table 3. NFL and GOSE. Modified Poisson Regression for 6-month GOSE

Supplementary Table 4. UCH-L1 and GOSE. Modified Poisson Regression

Supplementary Table 5. GFAP and GOSE. Modified Poisson Regression

Supplementary Table 6. Unadjusted relative risks (RRs) for the association between log biomarker concentrations and outcome

Supplementary Table 7. Adjusted relative risks (RRs) for the association between log biomarker concentrations and outcomes in patients with GCS=3 and GCS =15

Supplementary Table 8. Frequency of unfavourable outcomes (GOSE ≤4) within each biomarker concentration quintile for patients with GCS 3 and GCS 15

Supplementary Figure 1. CONSORT diagram for analysis sample

|  | CENTER-TBI core study  (4509) |  |
| --- | --- | --- |
|  |  | Age <16 (149/ 4509) |
|  | Age 16 or over  (4360) |  |
|  |  | GCS missing (163/ 4360) |
|  | Baseline GCS available  (4197) |  |
|  |  | GOSE at 6 months missing (655/ 4197) |
|  | Six-month GOSE available  (3542) |  |
|  |  | No biomarker sample in first 24 hrs (948/ 3542) |
|  | GCS 3-8 (629)  GCS 9-12 (222)  GCS 13-15 (1743) |  |
|  |  | CT unavailable (115/ 1743) |
|  | GCS 13-15 CT- (868)  GCS 13-15 CT+ (760) |  |
|  |  |  |

Supplementary Figure 2. Distributions of baseline GCS total scores. The graph shows the percentage of patients with each GCS score (N=2479).


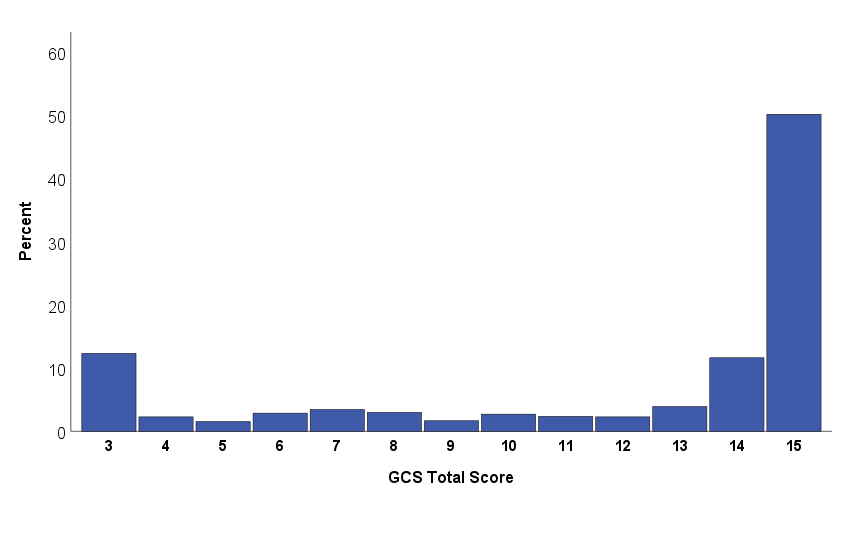


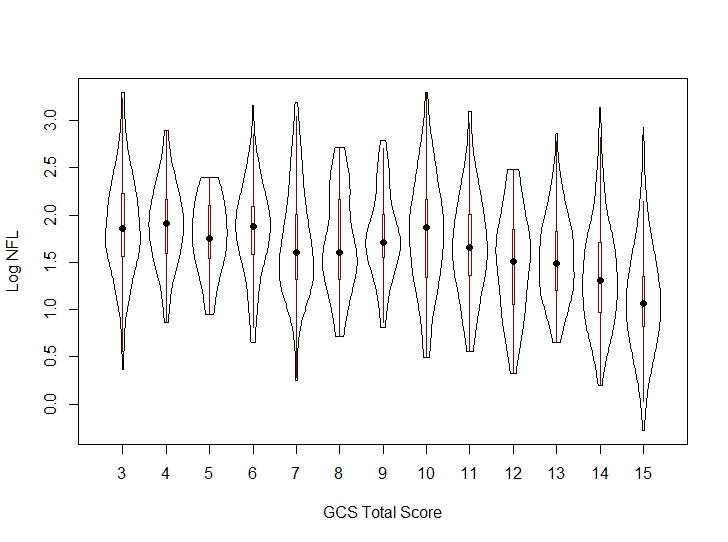


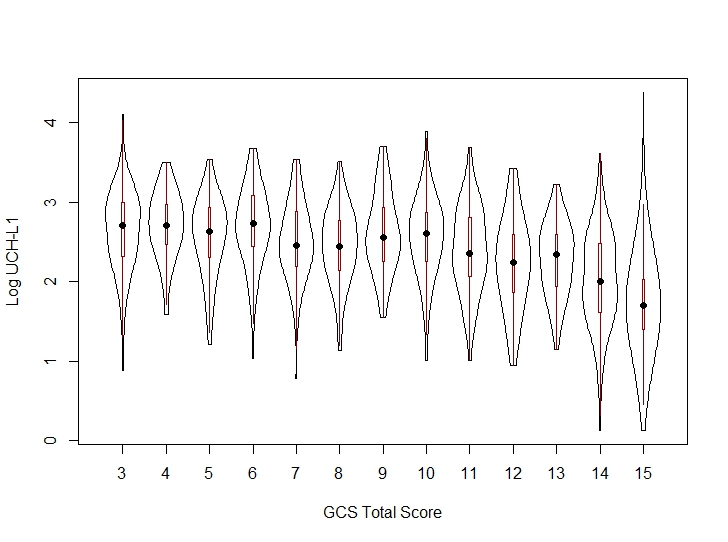


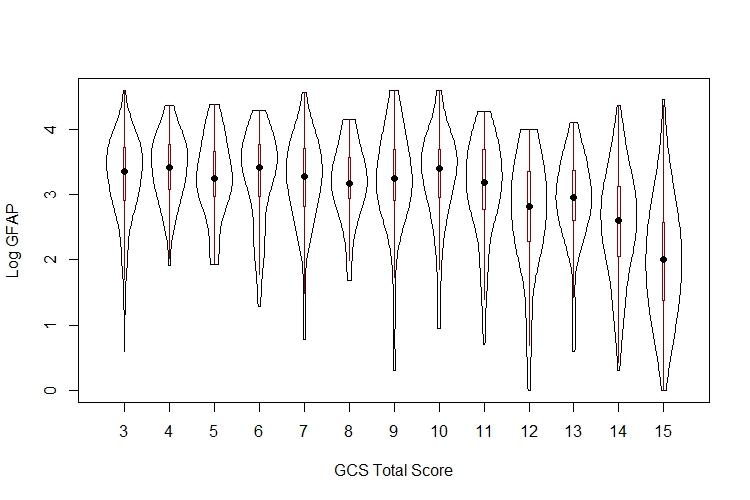


Supplementary Figure 3. Violin plots of log biomarker concentrations for each GCS total score (N=2479). The plots show the interquartile ranges (red boxes), medians (black dots), and density plots (outlines) of concentrations.

Supplementary Figure 4. Distributions of concentrations of log NFL, log UCH-L1 and log GFAP shown as density plots with embedded boxplots. Concentrations are displayed on a log 10 scale. Distributions are shown for (a) all patients (N=2479) (b) GCS 13-15 CT+ (N=868) and CT- (N=760), (c) GCS 3-8 (N=629) and 9-12 (N=222), (d) patients at GCS ceiling and floor (GCS=15 N=1243 and GCS=3 N=305). NFL= neurofilament protein-light; neurofilament protein-light, UCH-L1=ubiquitin carboxy-terminal hydrolase L1; GFAP=glial fibrillary acidic protein.

(a)

(c)

(d)

(b)

Supplementary Table 1. Number of samples (N), median, range (minimum and maximum value) and log 10 of the median for biomarker concentrations for each quintile within each GCS severity group (GCS 13-15 CT- N=868, GCS 13-15 CT+ N=760, GCS 9-12 N=222 and GCS 3-8 N=629)

|  |  | GCS 13-15 | | | | GCS 13-15 CT- | | | | GCS 13-15 CT+ | | | |
| --- | --- | --- | --- | --- | --- | --- | --- | --- | --- | --- | --- | --- | --- |
|  |  | N | Median | Range | Log10 | N | Median | Range | Log10 | N | Median | Range | Log10 |
| NFL | 1 | 325 | 4·20 | 0·53-6·09 | 0·62 | 172 | 3·40 | 0·53-4·77 | 0·53 | 152 | 6·98 | 1·25-10·3 | 0·84 |
| pg/mL | 2 | 326 | 7·93 | 6·12-10·1 | 0·90 | 174 | 5·91 | 4·78-7·19 | 0·77 | 151 | 13·0 | 10·3-16·4 | 1·11 |
|  | 3 | 326 | 13·0 | 10·16-17·0 | 1·11 | 175 | 8·77 | 7·21-10·8 | 0·94 | 153 | 21·5 | 16·4-28·3 | 1·33 |
|  | 4 | 326 | 23·6 | 17·1-34·9 | 1·37 | 173 | 14·2 | 10·9-19·4 | 1·15 | 152 | 39·7 | 28·4-58·8 | 1·60 |
|  | 5 | 325 | 65·4 | 35·1-1·37k | 1·82 | 174 | 30·4 | 19·5-1·38k | 1·48 | 152 | 104·5 | 59·2-1·28k | 2·02 |
|  | Total | 1628 |  |  |  | 868 |  |  |  | 760 |  |  |  |
| UCH-L1 | 1 | 324 | 12·5 | 1·34-23·5 | 1·10 | 172 | 8·81 | 1·34-14·8 | 0·94 | 152 | 26·0 | 1·34-44·0 | 1·42 |
| pg/mL | 2 | 325 | 33·1 | 23·6-45·0 | 1·52 | 173 | 23·8 | 14·9-30·1 | 1·38 | 152 | 60·6 | 44·1-82·1 | 1·78 |
|  | 3 | 325 | 59·0 | 45-81·2 | 1·77 | 173 | 37·9 | 30·1-47·4 | 1·58 | 152 | 111 | 82·3-162 | 2·05 |
|  | 4 | 325 | 116 | 81·2-194 | 2·06 | 173 | 61·7 | 47·4-85·8 | 1·79 | 152 | 234 | 162-332 | 2·37 |
|  | 5 | 324 | 356 | 195-24·1k | 2·55 | 172 | 148 | 86·1-23·2k | 2·17 | 152 | 512 | 333-24·1k | 2·71 |
|  | Total | 1623 |  |  |  | 863 |  |  |  | 760 |  |  |  |
| GFAP | 1 | 324 | 9 | 1·32-20 | 0·95 | 179 | 7 | 1·32-11 | 0·85 | 155 | 61 | 2-145 | 1·79 |
| pg/mL | 2 | 325 | 41 | 21-82 | 1·61 | 176 | 18·5 | 12-27 | 1·27 | 149 | 224 | 146-330 | 2·35 |
|  | 3 | 325 | 145 | 83-244 | 2·16 | 166 | 44 | 28-67 | 1·64 | 152 | 504·5 | 333-747 | 2·70 |
|  | 4 | 325 | 437 | 245-778 | 2·64 | 174 | 113 | 68-171 | 2·05 | 152 | 1·1k | 748-1·82k | 3·06 |
|  | 5 | 324 | 1·76k | 795-28·7k | 3·25 | 173 | 423 | 172-11·5k | 2·63 | 152 | 3·8k | 1·82k-28·7k | 3·58 |
|  | Total | 1628 |  |  |  | 868 |  |  |  | 760 |  |  |  |

Supplementary Table 1 (continued)

|  |  | GCS 9-12 | | |  | GCS 3-8 | |  |  |
| --- | --- | --- | --- | --- | --- | --- | --- | --- | --- |
|  |  | N | Median | Range | Log10 | N | Median | Range | Log10 |
| NFL | 1 | 44 | 8·01 | 2·08-17·0 | 0·90 | 126 | 15·8 | 1·77-25·5 | 1·20 |
| pg/mL | 2 | 45 | 25·6 | 17·7-37·0 | 1·41 | 125 | 36·2 | 26·0-46·5 | 1·56 |
|  | 3 | 44 | 48·2 | 38·0-62·2 | 1·68 | 126 | 63·5 | 46·7-88·3 | 1·80 |
|  | 4 | 45 | 85·7 | 62·9-142 | 1·93 | 126 | 123 | 89·0-191 | 2·09 |
|  | 5 | 44 | 268 | 146-2·0k | 2·43 | 126 | 302 | 193-2·0k | 2·48 |
|  | Total | 222 |  |  |  | 629 |  |  |  |
| UCH-L1 | 1 | 44 | 45·4 | 8·86-101 | 1·66 | 125 | 97·7 | 6·1-164 | 1·99 |
| pg/mL | 2 | 45 | 151 | 103-205 | 2·18 | 126 | 241 | 165-325 | 2·38 |
|  | 3 | 44 | 278 | 207-394 | 2·44 | 126 | 429 | 326-588 | 2·63 |
|  | 4 | 45 | 553 | 407-760 | 2·74 | 126 | 793 | 588-1·0k | 2·90 |
|  | 5 | 44 | 1323 | 764-7·8k | 3·12 | 125 | 1593 | 1·0k-13k | 3·20 |
|  | Total | 222 |  |  |  | 628 |  |  |  |
| GFAP | 1 | 45 | 106 | 1·32-344 | 2·03 | 125 | 257 | 4-618 | 2·41 |
| pg/mL | 2 | 44 | 697 | 387-956 | 2·84 | 127 | 1042 | 631-1·50k | 3·02 |
|  | 3 | 44 | 1601 | 963-2·23k | 3·20 | 125 | 2·23k | 1·50k-3·08k | 3·35 |
|  | 4 | 45 | 3·88k | 2·23k-5·51k | 3·59 | 126 | 4·21k | 3·09K-6·34k | 3·62 |
|  | 5 | 44 | 8·62k | 5·51k-40k | 3·94 | 126 | 9·66k | 6·47k-40k | 3·99 |
|  | Total | 222 |  |  |  | 629 |  |  |  |

NFL= neurofilament protein-light; neurofilament protein-light, UCH-L1=ubiquitin carboxy-terminal hydrolase L1; GFAP=glial fibrillary acidic protein.

Supplementary Table 2. Number of impaired scores on the dichotomised GOSE for each quintile band, with percentages and 95% confidence intervals in brackets, within each GCS severity group (GCS 13-15 CT- N=868, GCS 13-15 CT+ N=760, GCS 9-12 N=222 and GCS 3-8 N=629).

|  | **GCS 13-15 CT-** |  |  | **GCS 13-15 CT+** |  |  |
| --- | --- | --- | --- | --- | --- | --- |
| NFL quintile | GOSE<8 | GOSE≤6 | GOSE≤4 | GOSE<8 | GOSE≤6 | GOSE≤4 |
| 1 | 57 (33[26-40]) | 19 (11[6-16]) | 4 (2[0-5]) | 80 (53[45-61]) | 36 (24[17-30]) | 4 (3[0-5]) |
| 2 | 61 (35[28-42]) | 22 (13[8-18]) | 2 (1[0-3]) | 87 (58[50-66]) | 46 (30[23-38]) | 12 (8[4-12]) |
| 3 | 60 (34[27-41]) | 23 (13[8-18]) | 5 (3[0-5]) | 98 (64[56-72]) | 53 (35[27-42]) | 22 (14[9-20]) |
| 4 | 75 (43[36-51]) | 29 (17[11-22]) | 9 (5[2-9]) | 97 (64[56-71]) | 71 (47[39-55]) | 33 (22[15-28]) |
| **5** | 90 (52[44-59]) | 57 (33[26-40]) | 29 (17[11-22]) | 117 (77[70-84]) | 88 (58[50-66]) | 50 (33[25-40]) |
|  | **GCS 9-12** |  |  | **GCS 3-8** |  |  |
| NFL quintile | GOSE<8 | GOSE≤6 | GOSE≤4 | GOSE<8 | GOSE≤6 | GOSE≤4 |
| 1 | 29 (66[52-80]) | 19 (43[29-58]) | 9 (20[9-32]) | 106 (84[78-91]) | 77 (61[53-70]) | 37 (29[21-37]) |
| 2 | 31 (69[55-82]) | 20 (44[30-59]) | 11 (24[12-37]) | 114 (91[86-96]) | 98 (78[71-86]) | 60 (48[39-57]) |
| 3 | 37 (84[73-95]) | 34 (77[65-90]) | 18 (41[26-55]) | 117 (93[88-97]) | 108 (86[80-92]) | 74 (59[50-67]) |
| 4 | 37 (82[71-93]) | 35 (78[66-90]) | 23 (51[37-66]) | 118 (94[89-98]) | 113 (90[84-95]) | 83 (66[58-74]) |
| **5** | 40 (91[82-99]) | 38 (86[76-97]) | 31 (70[57-84]) | 123 (98[95-100]) | 122 (97[94-100]) | 108 (86[80-92]) |
|  | **GCS 13-15 CT-** |  |  | **GCS 13-15 CT+** |  |  |
| UCH-L1 quintile | GOSE<8 | GOSE≤6 | GOSE≤4 | GOSE<8 | GOSE≤6 | GOSE≤4 |
| 1 | 50 (29[22-36]) | 20 (12[7-16]) | 6 (3[1-6]) | 75 (49[41-57]) | 41 (27[20-34]) | 8 (5[2-9]) |
| 2 | 61 (35[28-42]) | 24 (14[9-19]) | 8 (5[1-8]) | 86 (57[49-64]) | 46 (30[23-38]) | 9 (6[2-10]) |
| 3 | 66 (38[31-45]) | 25 (14[9-20]) | 9 (5[2-9]) | 98 (64[57-72]) | 51 (34[26-41]) | 18 (12[7-17]) |
| 4 | 67 (39[31-46]) | 28 (16[11-22]) | 10 (6[2-9]) | 105 (69[62-76]) | 73 (48[40-56]) | 39 (26[19-33]) |
| **5** | 94 (55[47-62]) | 52 (30[23-37]) | 16 (9[5-14]) | 115 (76[69-82]) | 83 (55[47-63]) | 47 (31[24-38]) |
|  | **GCS 9-12** |  |  | **GCS 3-8** |  |  |
| UCH-L1 quintile | GOSE<8 | GOSE≤6 | GOSE≤4 | GOSE<8 | GOSE≤6 | GOSE≤4 |
| 1 | 30 (68[54-82]) | 20 (45[31-60]) | 9 (20[9-32]) | 107 (86[79-92]) | 79 (63[55-72]) | 43 (34[26-43]) |
| 2 | 34 (76[63-88]) | 23 (51[37-66]) | 12 (27[14-40]) | 109 (87[81-92]) | 98 (78[71-85]) | 49 (39[30-47]) |
| 3 | 32 (73[60-86]) | 29 (66[52-80]) | 14 (32[18-46]) | 115 (91[86-96]) | 101 (80[73-87]) | 69 (55[46-63]) |
| 4 | 36 (80[68-92]) | 34 (76[63-88]) | 23 (51[37-66]) | 125 (99[98-101]) | 120 (95[92-99]) | 92 (73[65-81]) |
| 5 | 42 (95[89-100]) | 40 (91[82-99]) | 34 (77[65-90]) | 121 (97[94-100]) | 119 (95[91-99]) | 108 (86[80-92]) |

Supplementary Table 2. (Continued)

|  | **GCS 13-15 CT-** |  |  | **GCS 13-15 CT+** |  |  |
| --- | --- | --- | --- | --- | --- | --- |
| GFAP quintile | GOSE<8 | GOSE≤6 | GOSE≤4 | GOSE<8 | GOSE≤6 | GOSE≤4 |
| 1 | 62 (35[28-42]) | 24 (13[8-18]) | 4 (2[0-4]) | 79 (51[43-59]) | 48 (31[24-38]) | 16 (10[6-15]) |
| 2 | 66 (38[30-45]) | 26 (15[10-20]) | 8 (5[1-8]) | 90 (60[53-68]) | 46 (31[23-38]) | 10 (7[3-11]) |
| 3 | 60 (36[29-43]) | 26 (16[10-21]) | 13 (8[4-12]) | 88 (58[50-66]) | 53 (35[27-42]) | 20 (13[8-19]) |
| 4 | 74 (43[35-50]) | 32 (18[13-24]) | 15 (9[4-13]) | 106 (70[62-77]) | 60 (39[32-47]) | 23 (15[9-21]) |
| **5** | 81 (47[39-54]) | 42 (24[18-31]) | 9 (5[2-9]) | 116 (76[70-83]) | 87 (57[49-65]) | 52 (34[27-42]) |
|  | **GCS 9-12** |  |  | **GCS 3-8** |  |  |
| GFAP quintile | GOSE<8 | GOSE≤6 | GOSE≤4 | GOSE<8 | GOSE≤6 | GOSE≤4 |
| 1 | 31 (69[55-82]) | 20 (44[30-59]) | 9 (20[8-32]) | 107 (86[79-92]) | 85 (68[60-76]) | 51 (41[32-49]) |
| 2 | 32 (73[60-86]) | 27 (61[47-76]) | 14 (32[18-46]) | 114 (90[84-95]) | 95 (75[67-82]) | 48 (38[29-46]) |
| 3 | 32 (73[60-86]) | 24 (55[40-69]) | 13 (30[16-43]) | 111 (89[83-94]) | 102 (82[75-88]) | 64 (51[42-60]) |
| 4 | 38 (84[74-95]) | 35 (78[66-90]) | 19 (42[28-57]) | 121 (96[93-99]) | 114 (90[85-96]) | 92 (73[65-81]) |
| 5 | 41 (93[86-100]) | 40 (91[82-99]) | 37 (84[73-95]) | 125 (99[98-100]) | 122 (97[94-100]) | 107 (85[79-91]) |

Supplementary Table 3. NFL and GOSE. Modified Poisson Regression for 6-month GOSE in four severity groups, including age, sex, major extracranial injury (MEI), time to sample, and log NFL concentration. The table shows relative risks (RRs) with 95% CIs and p values.

|  | **GCS 13-15 CT-** |  |  |  |  |  | **GCS 13-15 CT+** |  |  |  |  |  |
| --- | --- | --- | --- | --- | --- | --- | --- | --- | --- | --- | --- | --- |
| NFL | GOSE<8 |  | GOSE≤6 |  | GOSE≤4 |  | GOSE<8 |  | GOSE≤6 |  | GOSE≤4 |  |
|  | RR (95% CIs) | p | RR (95% CIs) | p | RR (95% CIs) | p | RR (95% CIs) | p | RR (95% CIs) | p | RR (95% CIs) | p |
| Age | 1·01 (0·97-1·06) | ·56 | 1·06 (0·98-1·14) | ·15 | 1·32 (1·11-1·57) | ·0018 | 1·03 (1·00-1·06) | ·064 | 1·04 (0·99-1·09) | ·12 | 1·39 (1·25-1·56) | <·0001 |
| Sex (M vs F) | 0·72 (0·61-0·84) | ·0001 | 0·76 (0·56-1·01) | ·061 | 0·83 (0·46-1·50) | ·53 | 0·95 (0·85-1·06) | ·33 | 0·95 (0·79-1·14) | ·56 | 0·97 (0·71-1·32) | ·84 |
| MEI (Y vs N) | 1·62 (1·37-1·92) | <·0001 | 1·84 (1·35-2·51) | ·0001 | 1·98 (1·05-3·71) | ·034 | 1·23 (1·11-1·37) | ·0001 | 1·22 (1·02-1·46) | ·029 | 1·20 (0·88-1·65) | ·25 |
| Sampling time |  |  |  |  |  |  |  |  |  |  |  |  |
| >8-16h vs 0-8h | 1·44 (1·18-1·75) | ·0003 | 1·57 (1·10-2·24) | ·013 | 0·94 (0·44-1·98) | ·86 | 1·24 (1·07-1·44) | ·0052 | 1·04 (0·79-1·35) | ·80 | 1·06 (0·67-1·66) | ·81 |
| >16-24h vs 0-8h | 1·29 (1·06-1·58) | ·013 | 1·27 (0·88-1·84) | ·21 | 1·33 (0·71-2·52) | ·37 | 1·02 (0·88-1·18) | ·79 | 1·16 (0·92-1·46) | ·21 | 1·08 (0·75-1·55) | ·70 |
| Log NFL | 1·28 (1·05-1·58) | ·016 | 1·97 (1·43-2·73) | <·0001 | 3·72 (2·47-5·60) | <·0001 | 1·29 (1·15-1·44) | <·0001 | 1·72 (1·44-2·04) | <·0001 | 2·81 (2·14-3·68) | <·0001 |
|  | **GCS 9-12** |  |  |  |  |  | **GCS 3-8** |  |  |  |  |  |
|  | GOSE<8 |  | GOSE≤6 |  | GOSE≤4 |  | GOSE<8 |  | GOSE≤6 |  | GOSE≤4 |  |
|  | RR (95% CIs) | P | RR (95% CIs) | P | RR (95% CIs) | P | RR (95% CIs) | P | RR (95% CIs) | P | RR (95% CIs) | P |
| Age | 1·10 (1·06-1·15) | <·0001 | 1·12 (1·07-1·18) | <·0001 | 1·35 (1·23-1·48) | <·0001 | 1·02 (1·01-1·03) | ·0034 | 1·02 (1·00-1·04) | ·049 | 1·11 (1·07-1·14) | <·0001 |
| Sex (M vs F) | 1·06 (0·92-1·24) | ·43 | 1·25 (1·00-1·56) | ·046 | 1·33 (0·97-1·82) | ·076 | 1·00 (0·95-1·05) | ·99 | 0·99 (0·92-1·07) | ·79 | 0·96 (0·83-1·09) | ·50 |
| MEI (Y vs N) | 1·13 (1·00-1·27) | ·055 | 1·13 (0·95-1·34) | ·17 | 0·73 (0·54-0·98) | ·039 | 1·03 (0·98-1·09) | ·20 | 1·09 (1·01-1·17) | ·036 | 1·20 (1·05-1·38) | ·0064 |
| Sampling time |  |  |  |  |  |  |  |  |  |  |  |  |
| >8-16h vs 0-8h | 1·08 (0·90-1·29) | ·42 | 0·98 (0·77-1·25) | ·90 | 1·10 (0·74-1·64) | ·65 | 1·03 (0·97-1·10) | ·36 | 1·04 (0·94-1·14) | ·46 | 1·03 (0·87-1·21) | ·75 |
| >16-24h vs 0-8h | 0·94 (0·80-1·11) | ·45 | 0·93 (0·75-1·16) | ·52 | 1·16 (0·82-1·65) | ·40 | 0·99 (0·93-1·05) | ·73 | 0·96 (0·87-1·05) | ·38 | 0·94 (0·80-1·11) | ·46 |
| Log NFL | 1·11 (0·97-1·26) | ·14 | 1·39 (1·16-1·67) | ·0003 | 1·78 (1·40-2·28) | <·0001 | 1·09 (1·04-1·14) | ·0003 | 1·29 (1·20-1·39) | <·0001 | 1·68 (1·50-1·89) | <·0001 |

Supplementary Table 4. UCH-L1 and GOSE. Modified Poisson Regression for 6-month GOSE in four severity groups, including age, sex, major extracranial injury(MEI), time to sample, and log UCH-L1 concentration. The table shows relative risks (RRs) with 95% CIs and p values.

|  | **GCS 13-15 CT-** |  |  |  |  |  | **GCS 13-15 CT+** |  |  |  |  |  |
| --- | --- | --- | --- | --- | --- | --- | --- | --- | --- | --- | --- | --- |
|  | GOSE<8 |  | GOSE≤6 |  | GOSE≤4 |  | GOSE<8 |  | GOSE≤6 |  | GOSE≤4 |  |
|  | RR (95% CIs) | p | RR (95% CIs) | p | RR (95% CIs) | p | RR (95% CIs) | p | RR (95% CIs) | p | RR (95% CIs) | p |
| Age | 1·04 (1·00-1·08) | ·082 | 1·12 (1·04-1·21) | ·0017 | 1·53 (1·27-1·85) | <·0001 | 1·04 (1·01-1·07) | ·011 | 1·06 (1·01-1·11) | ·015 | 1·44 (1·28-1·61) | <·0001 |
| Sex (M vs F) | 0·71 (0·61-0·84) | ·0001 | 0·75 (0·56-1·01) | ·054 | 0·85 (0·46-1·57) | ·61 | 0·96 (0·86-1·07) | ·44 | 0·97 (0·81-1·16) | ·73 | 1·01 (0·74-1·38) | ·94 |
| MEI (Y vs N) | 1·57 (1·32-1·88) | <·0001 | 1·74 (1·27-2·39) | ·0006 | 2·01 (1·17-3·47) | ·012 | 1·19 (1·07-1·32) | ·0016 | 1·17 (0·98-1·40) | ·093 | 1·00 (0·72-1·39) | ·99 |
| Sampling time |  |  |  |  |  |  |  |  |  |  |  |  |
| >8-16h vs 0-8h | 1·44 (1·19-1·75) | ·0002 | 1·69 (1·19-2·39) | ·0031 | 1·03 (0·49-2·16) | ·95 | 1·29 (1·12-1·49) | ·0005 | 1·12 (0·87-1·46) | ·38 | 1·21 (0·77-1·88) | ·41 |
| >16-24h vs 0-8h | 1·30 (1·06-1·59) | ·011 | 1·34 (0·93-1·93) | ·12 | 1·63 (0·88-3·00) | ·12 | 1·10 (0·95-1·26) | ·20 | 1·36 (1·09-1·70) | ·0058 | 1·35 (0·93-1·96) | ·11 |
| Log UCH-L1 | 1·28 (1·09-1·51) | ·0031 | 1·71 (1·29-2·26) | ·0002 | 1·84 (1·09-3·12) | ·023 | 1·33 (1·21-1·47) | <·0001 | 1·64 (1·40-1·91) | <·0001 | 2·92 (2·19-3·90) | <·0001 |
|  |  |  |  |  |  |  |  |  |  |  |  |  |
|  | **GCS 9-12** |  |  |  |  |  | **GCS 3-8** |  |  |  |  |  |
|  | GOSE<8 |  | GOSE≤6 |  | GOSE≤4 |  | GOSE<8 |  | GOSE≤6 |  | GOSE≤4 |  |
|  | RR (95% CIs) | P | RR (95% CIs) | P | RR (95% CIs) | P | RR (95% CIs) | P | RR (95% CIs) | P | RR (95% CIs) | P |
| Age | 1·10 (1·06-1·14) | <·0001 | 1·12 (1·07-1·18) | <·0001 | 1·35 (1·23-1·48) | <·0001 | 1·02 (1·01-1·03) | ·0014 | 1·02 (1·01-1·04) | ·0093 | 1·12 (1·08-1·15) | <·0001 |
| Sex (M vs F) | 1·06 (0·91-1·23) | ·44 | 1·23 (0·99-1·54) | ·067 | 1·26 (0·91-1·73) | ·16 | 1·00 (0·95-1·06) | ·96 | 0·99 (0·92-1·07) | ·80 | 0·96 (0·84-1·10) | ·57 |
| MEI (Y vs N) | 1·13 (1·00-1·27) | ·053 | 1·16 (0·98-1·38) | ·094 | 0·77 (0·58-1·04) | ·084 | 1·02 (0·98-1·08) | ·35 | 1·06 (0·99-1·15) | ·11 | 1·17 (1·02-1·34) | ·024 |
| Sampling time |  |  |  |  |  |  |  |  |  |  |  |  |
| >8-16h vs 0-8h | 1·08 (0·91-1·28) | ·40 | 0·99 (0·79-1·24) | ·92 | 1·04 (0·72-1·51) | ·85 | 1·06 (0·99-1·13) | ·090 | 1·11 (1·00-1·22) | ·047 | 1·13 (0·96-1·33) | ·15 |
| >16-24h vs 0-8h | 0·94 (0·80-1·11) | ·47 | 0·92 (0·75-1·13) | ·43 | 1·12 (0·81-1·56) | ·48 | 1·04 (0·97-1·11) | ·28 | 1·07 (0·97-1·18) | ·19 | 1·12 (0·96-1·31) | ·15 |
| Log UCH-L1 | 1·16 (1·04-1·30) | ·011 | 1·49 (1·27-1·76) | <·0001 | 2·02 (1·58-2·58) | <·0001 | 1·12 (1·06-1·19) | ·0001 | 1·36 (1·25-1·48) | <·0001 | 1·94 (1·68-2·24) | <·0001 |

Supplementary Table 5. GFAP and GOSE. Modified Poisson Regression for 6-month GOSE in four severity groups, including age, sex, major extracranial injury (MEI), time to sample, and log GFAP concentration. The table shows relative risks (RRs) with 95% CIs and p values.

|  | **GCS 13-15 CT-** |  |  |  |  |  | **GCS 13-15 CT+** |  |  |  |  |  |
| --- | --- | --- | --- | --- | --- | --- | --- | --- | --- | --- | --- | --- |
|  | GOSE<8 |  | GOSE≤6 |  | GOSE≤4 |  | GOSE<8 |  | GOSE≤6 |  | GOSE≤4 |  |
|  | RR (95% CIs) | p | RR (95% CIs) | p | RR (95% CIs) | p | RR (95% CIs) | p | RR (95% CIs) | p | RR (95% CIs) | p |
| Age | 1·04 (1·00-1·08) | ·052 | 1·14 (1·06-1·22) | ·0006 | 1·55 (1·29-1·87) | <·0001 | 1·04 (1·01-1·07) | ·0060 | 1·07 (1·02-1·12) | ·0071 | 1·48 (1·32-1·66) | <·0001 |
| Sex (M vs F) | 0·72 (0·61-0·84) | <·0001 | 0·75 (0·56-1·01) | ·055 | 0·88 (0·47-1·63) | ·68 | 0·95 (0·85-1·06) | ·39 | 0·96 (0·80-1·15) | ·63 | 1·02 (0·75-1·39) | ·91 |
| MEI (Y vs N) | 1·73 (1·46-2·05) | <·0001 | 2·09 (1·52-2·87) | <·0001 | 2·61 (1·45-4·72) | ·0014 | 1·23 (1·10-1·36) | ·0001 | 1·24 (1·03-1·48) | ·021 | 1·22 (0·89-1·67) | ·21 |
| Sampling time |  |  |  |  |  |  |  |  |  |  |  |  |
| >8-16h vs 0-8h | 1·45 (1·19-1·76) | ·0003 | 1·60 (1·13-2·28) | ·0089 | 0·95 (0·45-2·02) | ·90 | 1·24 (1·07-1·44) | ·0054 | 1·04 (0·80-1·37) | ·76 | 0·95 (0·62-1·47) | ·82 |
| >16-24h vs 0-8h | 1·33 (1·09-1·63) | ·0056 | 1·41 (0·98-2·02) | ·064 | 1·49 (0·79-2·80) | ·22 | 1·04 (0·90-1·21) | ·59 | 1·22 (0·97-1·54) | ·091 | 1·00 (0·70-1·42) | ·99 |
| Log GFAP | 1·00 (0·88-1·14) | ·99 | 1·07 (0·85-1·35) | ·58 | 1·03 (0·69-1·54) | ·89 | 1·21 (1·12-1·31) | <·0001 | 1·36 (1·19-1·56) | <·0001 | 2·14 (1·69-2·70) | <·0001 |
|  |  |  |  |  |  |  |  |  |  |  |  |  |
|  | **GCS 9-12** |  |  |  |  |  | **GCS 3-8** |  |  |  |  |  |
|  | GOSE<8 |  | GOSE≤6 |  | GOSE≤4 |  | GOSE<8 |  | GOSE≤6 |  | GOSE≤4 |  |
|  | RR (95% CIs) | P | RR (95% CIs) | P | RR (95% CIs) | P | RR (95% CIs) | P | RR (95% CIs) | P | RR (95% CIs) | P |
| Age | 1·10 (1·06-1·15) | <·0001 | 1·13 (1·07-1·19) | <·0001 | 1·36 (1·23-1·50) | <·0001 | 1·02 (1·01-1·03) | ·0011 | 1·03 (1·01-1·04) | ·0075 | 1·12 (1·09-1·16) | <·0001 |
| Sex (M vs F) | 1·07 (0·92-1·25) | ·41 | 1·26 (1·01-1·58) | ·041 | 1·33 (0·97-1·84) | ·081 | 1·00 (0·95-1·06) | ·93 | 0·99 (0·92-1·07) | ·87 | 0·97 (0·84-1·11) | ·64 |
| MEI (Y vs N) | 1·13 (1·00-1·28) | ·050 | 1·15 (0·96-1·36) | ·12 | 0·75 (0·56-1·00) | ·052 | 1·04 (0·99-1·09) | ·13 | 1·11 (1·02-1·19) | ·010 | 1·26 (1·10-1·44) | ·0008 |
| Sampling time |  |  |  |  |  |  |  |  |  |  |  |  |
| >8-16h vs 0-8h | 1·06 (0·88-1·27) | ·54 | 0·97 (0·76-1·23) | ·78 | 1·11 (0·75-1·64) | ·61 | 1·02 (0·96-1·09) | ·48 | 1·03 (0·93-1·13) | ·59 | 1·01 (0·86-1·20) | ·87 |
| >16-24h vs 0-8h | 0·95 (0·80-1·11) | ·50 | 0·92 (0·75-1·14) | ·46 | 1·11 (0·80-1·54) | ·54 | 0·99 (0·93-1·05) | ·74 | 0·97 (0·88-1·06) | ·46 | 0·93 (0·80-1·10) | ·41 |
| Log GFAP | 1·07 (0·98-1·17) | ·12 | 1·28 (1·11-1·48) | ·0006 | 1·55 (1·23-1·95) | ·0002 | 1·09 (1·04-1·13) | ·0001 | 1·25 (1·16-1·34) | <·0001 | 1·56 (1·38-1·77) | <·0001 |

Supplementary Table 6. Unadjusted relative risks (RRs), with 95% confidence intervals in brackets, for the association between log biomarker concentrations and outcomes within each GCS severity group (GCS 13-15 CT- N=868, GCS 13-15 CT+ N=760, GCS 9-12 N=222 and GCS 3-8 N=629).

|  |  |  | RR (95% CI) | P |  | RR (95% CI) | | P |
| --- | --- | --- | --- | --- | --- | --- | --- | --- |
|  |  |  | |  |  | | |  |
|  |  | GCS 13-15 CT- | |  | GCS 13-15 CT+ | | |  |
| GOSE<8 | Log NFL |  | 1·58 (1·34-1·86) | <·0001 |  | 1·33 (1·20-1·48) | | <·0001 |
|  | Log UCH-L1 |  | 1·44 (1·23-1·69) | <·0001 |  | 1·37 (1·24-1·50) | | <·0001 |
|  | Log GFAP |  | 1·18 (1·05-1·33) | ·0065 |  | 1·23 (1·14-1·33) | | <·0001 |
| GOSE≤6 | Log NFL |  | 2·60 (2·01-3·36) | <·0001 |  | 1·83 (1·55-2·17) | | <·0001 |
|  | Log UCH-L1 |  | 2·01 (1·55-2·60) | <·0001 |  | 1·69 (1·44-1·99) | | <·0001 |
|  | Log GFAP |  | 1·36 (1·10-1·67) | ·0046 |  | 1·42 (1·24-1·63) | | <·0001 |
| GOSE≤4 | Log NFL |  | 5·88 (4·22-8·20) | <·0001 |  | 3·55 (2·75-4·59) | | <·0001 |
|  | Log UCH-L1 |  | 2·31 (1·46-3·67) | ·0004 |  | 3·51 (2·70-4·55) | | <·0001 |
|  | Log GFAP |  | 1·44 (1·03-2·00) | ·031 |  | 2·39 (1·83-3·12) | | <·0001 |
|  |  |  |  |  |  | |  |  |
|  |  | GCS 9-12 | |  | GCS 3-8 | | |  |
| GOSE<8 | Log NFL |  | 1·24 (1·08-1·42) | ·0022 |  | 1·10 (1·05-1·16) | | ·0001 |
|  | Log UCH-L1 |  | 1·28 (1·12-1·45) | ·0002 |  | 1·13 (1·06-1·19) | | <·0001 |
|  | Log GFAP |  | 1·14 (1·03-1·26) | ·009 |  | 1·09 (1·05-1·14) | | ·0001 |
| GOSE≤6 | Log NFL |  | 1·60 (1·33-1·91) | <·0001 |  | 1·31 (1·22-1·41) | | <·0001 |
|  | Log UCH-L1 |  | 1·69 (1·44-2·00) | <·0001 |  | 1·37 (1·26-1·49) | | <·0001 |
|  | Log GFAP |  | 1·40 (1·21-1·63) | <·0001 |  | 1·26 (1·17-1·35) | | <·0001 |
| GOSE≤4 | Log NFL |  | 2·25 (1·73-2·93) | <·0001 |  | 1·80 (1·60-2·03) | | <·0001 |
|  | Log UCH-L1 |  | 2·60 (2·00-3·38) | <·0001 |  | 2·04 (1·76-2·36) | | <·0001 |
|  | Log GFAP |  | 1·95 (1·46-2·60) | <·0001 |  | 1·64 (1·43-1·87) | | <·0001 |

NFL= neurofilament protein-light; neurofilament protein-light, UCH-L1=ubiquitin carboxy-terminal hydrolase L1; GFAP=glial fibrillary acidic protein.

Supplementary Table 7. Adjusted relative risks (RRs), with 95% confidence intervals in brackets, for the association between log biomarker concentrations and outcomes in patients with GCS=3 (N=305) and GCS =15 (N=1243). The RRs have been adjusted for age, sex, major extracranial injury, and time to sample.

|  | GCS 3 |  | GCS 15 | P |
| --- | --- | --- | --- | --- |
|  | RR (95% CI) | P | RR (95% CI) |  |
| Log NFL | 1·54 (1·34-1·77) | <·0001 | 3·79 (2·65-5·40) | <·0001 |
| Log UCH-L1 | 1·80 (1·50-2·16) | <·0001 | 2·61 (1·92-3·55) | <·0001 |
| Log GFAP | 1·51 (1·29-1·77) | <·0001 | 1·82 (1·43-2·34) | <·0001 |

NFL= neurofilament protein-light; neurofilament protein-light, UCH-L1=ubiquitin carboxy-terminal hydrolase L1; GFAP=glial fibrillary acidic protein.

Supplementary Table 8. Frequency of unfavourable outcomes (GOSE ≤4) within each biomarker concentration quintile for patients with GCS 3 (N=1243) and GCS 15 (N=305), with percentages and 95% CIs in brackets, and the number of patients in each quintile. The concentration limits used to allocate values to quintiles for patients with GCS 3 and GCS 15 are from the GCS 3-8 and GCS 13-15 groups, respectively.

|  |  | NFL | N per | UCH-L1 | N per | GFAP | N per |
| --- | --- | --- | --- | --- | --- | --- | --- |
|  | Quintile | N (% [95% CIs]) | quintile | N (% [95% CIs]) | quintile | N (% [95% CIs]) | quintile |
| GCS 3 | 1 | 19 (40[26-54]) | 47 | 17 (37[24-49]) | 46 | 29 (51[38-64]) | 57 |
|  | 2 | 25 (47[34-61]) | 53 | 23 (40[28-53]) | 57 | 21 (37[24-49]) | 57 |
|  | 3 | 37 (60[47-72]) | 62 | 36 (63[51-75]) | 57 | 34 (57[44-69]) | 60 |
|  | 4 | 49 (71[60-82]) | 69 | 51 (76[66-86]) | 67 | 52 (79[69-89]) | 66 |
|  | 5 | 67 (91[84-97]) | 74 | 70 (90[82-97]) | 78 | 61 (94[88-100]) | 65 |
|  |  |  |  |  |  |  |  |
| GCS 15 | 1 | 5 (2[0-3]) | 277 | 11 (4[2-6]) | 287 | 9 (3[1-5]) | 288 |
|  | 2 | 6 (2[0-4]) | 278 | 15 (5[3-8]) | 276 | 21 (7[4-10]) | 295 |
|  | 3 | 13 (5[2-7]) | 267 | 14 (5[3-8]) | 264 | 22 (8[5-12]) | 263 |
|  | 4 | 22 (9[5-13]) | 241 | 18 (7[4-11]) | 242 | 12 (5[2-8]) | 227 |
|  | 5 | 51 (28[22-35]) | 180 | 39 (23[17-29]) | 169 | 33 (19[14-25]) | 170 |

NFL= neurofilament protein-light; neurofilament protein-light, UCH-L1=ubiquitin carboxy-terminal hydrolase L1; GFAP=glial fibrillary acidic protein.
